# Nivolumab-Induced Autoimmune-Like Cholestatic Hepatitis in a Liver Transplant Recipient

**DOI:** 10.14309/crj.0000000000000416

**Published:** 2020-07-14

**Authors:** Chimaobi Anugwom, Thomas Leventhal

**Affiliations:** 1 Division of Gastroenterology, Hepatology and Nutrition, University of Minnesota, Minneapolis, MN

## Abstract

Cancer treatment has taken giant strides in recent years with the advent of immunotherapy, including checkpoint inhibitors. The use of these medications in liver transplant recipients has been debated, and the added effect of previous hepatitis C infection on the immune system in this setting, is poorly understood. We present a case of cholestatic hepatitis after the treatment of recurrent hepatocellular carcinoma with nivolumab in the post-transplant period. Understanding the pathophysiology is relevant to improving the management of this type of liver injury and expanding our knowledge of programmed death-1 inhibitors in liver transplant recipients.

## INTRODUCTION

The treatment of cancer has taken giant strides in recent years with the advent of immunotherapy.^1^ The checkpoint inhibitors have been approved for the treatment of metastatic melanoma, nonsmall cell lung cancer, and hepatocellular carcinoma (HCC).^2^ Nivolumab—a PD-1 (programmed death-1) inhibitor, has demonstrated significant survival benefit but has been associated with a myriad of immune-related toxicities. We present a case of cholestatic hepatitis after nivolumab treatment of recurrent HCC in a patient with a history of hepatitis C virus (HCV) cirrhosis.

## CASE REPORT

A 62-year-old man presented with a medical history of alcohol use disorder and chronic hepatitis C infection, with resultant cirrhosis that was further complicated by the development of HCC approximately 2 years before liver transplantation. His malignancy was detected through routine screening ultrasounds, and his serum alpha-feto protein was noted to be only mildly elevated during his disease course and most times within the normal range. He underwent deceased donor liver transplantation approximately 5 years before presentation, was cured of chronic hepatitis C using direct-acting antivirals (DAAs), and was on stable tacrolimus immunosuppressive monotherapy with excellent allograft function. After the removal of the explant, large tumor burden was noted, with microvascular and macrovascular invasion, but no adenopathy or evidence of extrahepatic spread. His Risk Estimation of Tumor Recurrence After Transplant score at the time of transplant was 5, suggesting >75% risk of recurrence. Given his high risk, he was checked every 3–6 months post-transplant period. Approximately 1 year after transplant, he was found to have a nodule in his right upper lung lobe and underwent resection, which demonstrated metastatic HCC. He developed further pulmonary nodules, and an endoscopic ultrasound-guided biopsy of a nodule in the left lower lung demonstrated poorly differentiated nonsmall cell lung cancer. Immunohistochemistry for this biopsy demonstrated “no programmed death ligand-1 expression.” Approximately 1 month after discovering this neoplasm, a mass on his abdominal wall was biopsied and demonstrated poorly differentiated nonsmall cell carcinoma.

He underwent several chemotherapy regimens including sorafenib, carboplatin/gemcitabine, combination folinic acid, fluorouracil, and oxaliplatin—all with minimal impact and progression of tumor burden on surveillance imaging. Approximately 2 months before presentation, he was started on systemic nivolumab, with palliative intent and radiation therapy for the abdominal wall metastasis. He was seen regularly in clinic for routine laboratory monitoring.

At his clinic visit, he was noted to have mild abdominal pain and severe jaundice but no fever, chills, nausea, or vomiting. Initial laboratory work was significant for elevated serum alkaline phosphatase, aspartate aminotransaminase, alanine aminotransaminase, and total bilirubin; and so, he was hospitalized.

**Figure 1.**
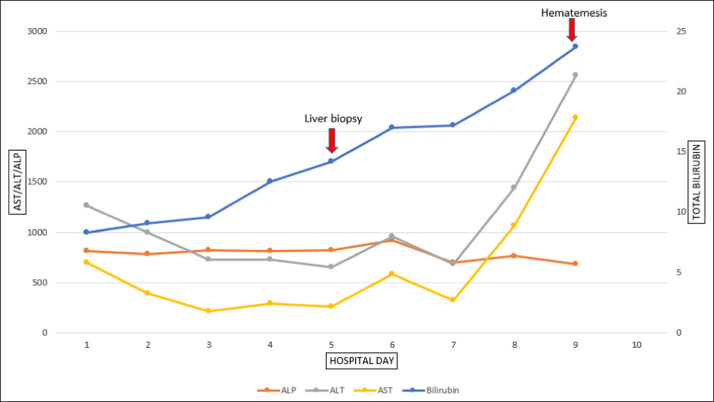
Graph showing the trend of liver tests (aminotransaminase, alanine aminotransaminase, alkaline phosphatase, and bilirubin) during hospitalization.

**Figure 2.**
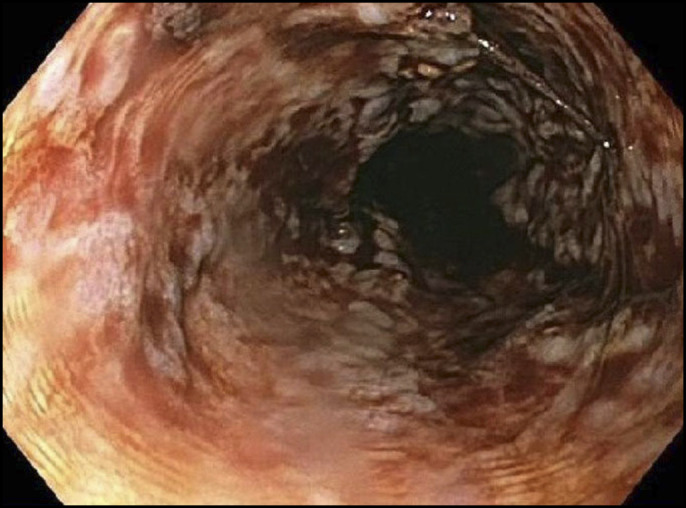
Upper endoscopic findings in the esophagus after an episode of massive hematemesis.

On the day of admission, he had an alkaline phosphatase of 813 IU/L, alanine aminotransaminase of 1,265 IU/L, aspartate aminotransaminase of 696 IU/L, and bilirubin of 8.3 mg/dL. He remained hemodynamically stable during the initial course of his hospitalization. Thoracic, abdominal, and pelvic computed tomography was negative for new liver or intra-abdominal masses but showed mild ascites. Given the concern for nivolumab-induced liver injury, he was started on high-dose intravenous steroids at 2 mg/kg daily and had relative temporary improvement in his liver tests. Additional evaluation for other causes of acute liver injury was negative for acute viral hepatitis, ischemic injury, and autoimmune hepatitis. Given the liver transplant status, acute cellular rejection was also considered in the differential, although felt unlikely, given the time from transplant and historically stable tacrolimus trough levels. A diagnostic paracentesis was performed in the setting of new ascites and demonstrated a serum-ascites albumin gradient of 1.1, an ascites protein of 2.0, and cell count with differential consistent with spontaneous bacterial peritonitis (SBP). He was started on appropriate therapy for SBP on day 2 of hospitalization. A transjugular liver biopsy was performed on day 5 of hospitalization to confirm the diagnosis of immune-induced liver injury and assess whether high-dose steroid therapy was effective. By day 7 of his hospital stay, his serum transaminases and bilirubin doubled and continued to increase precipitously (Figure 1). Ultimately, he developed a massive gastrointestinal bleed with hematemesis and hemodynamic instability, necessitating intubation for airway control and admission to the intensive care unit. An emergent upper endoscopy demonstrated severe esophagitis with denudation of the esophageal mucosa and gastric blood and clot precluding the completion of the endoscopic procedure (Figure 2). On admission, his intravenous steroids were increased to 4 mg/kg, given the concern of worsening immune-induced hepatitis. After a goals of care conference, his family agreed to pursue comfort cares, and he died within minutes of implementing comfort care measures.

**Figure 3.**
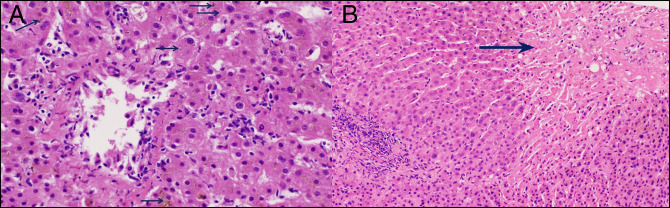
Liver biopsy specimen showing lobular inflammation with (A) associated perivenular necrosis and cholestasis (arrows) (hematoxylin and eosin stain, 400× magnification) and (B) associated perivenular necrosis (arrow) (hematoxylin and eosin stain, 200× magnification).

The patient's liver biopsy was reviewed by 2 pathologists, and this revealed lobular inflammation with perivenular necrosis and cholestasis consistent with nivolumab-induced cholestatic liver injury (Figure 3). There was no typical portal inflammation, ductopenia, ductulitis, or endotheliitis associated with any form of acute cellular, ductopenic, or antibody-mediated rejection.

## DISCUSSION

Nivolumab, a PD-1 inhibitor, has been approved by the Food and Drug Administration for treatment of nonsmall cell lung cancer, melanoma, renal cell carcinoma, and HCC based on trials highlighting the survival benefits and low rates of toxicity.^2–5^ Tumor cells escape immune surveillance by expressing the PD-1 ligand that binds to the PD-1 receptor on immune cells. This serves as a checkpoint of T-cell-mediated cell death.^6^ Hence, by targeting this PD-1/programmed death ligand-1 interaction, nivolumab—a human anti-PD-1 monoclonal antibody, can turn on T-cell-mediated antitumor effect.^7^ As expected, increasing the activity of the immune system results in a host of inflammatory adverse effects. These have been postulated to be immune-related and can affect the gastrointestinal tract, skin, cardiopulmonary system, and the liver.^8^

A wide spectrum of nivolumab-induced liver injury has been documented in the literature, with mostly transient forms reported.^9^ It is quite apparent that nivolumab produces an array of liver damage from hepatocellular pattern of disease to a handful of cholestatic liver injury.^9–11^ There is paucity of data on the use of checkpoint inhibitors in post-transplant patients for the treatment of metastatic cancer, and its efficacy and safety still require more studies. Case reports have reported high rates of graft loss or acute rejection after liver transplant, whereas some studies have shown a largely uneventful course, suggesting a feasibility of treatment with close surveillance and monitoring of liver function.^12–15^

In patients treated for hepatitis C infection, there may be a more complex underlying immunologic process. This immunomodulatory effect of the HCV is well-characterized but poorly understood, and the paradigm shift of the treatment of HCV from interferon to the direct-acting antivirals has provided an opportunity to evaluate the effect of HCV on the immune system. HCV escapes immune surveillance mainly by “switching off” the immune system, thus reducing the activity of cells that are responsible for the elimination of virus-infected cells.^16^ Chronic HCV also results in impaired function of CD8+ cells with increased expression of inhibiting receptors such as PD-1 and weak expression of interleukin 7 receptor.^17^ Once HCV replication is halted, there is an immune system reconstitution with normalization of natural killer cells and the activity of CD4+ and CD8+ cells.^18^ In our patient, the addition of a checkpoint inhibitor to an already altered immune system may be responsible for the increased activity of cytotoxic cells and severe hepatitis. Corticosteroids are the mainstay of therapy for nivolumab-induced liver injury; however, the response to treatment is variable. Severe or worsening disease may require the addition of mycophenolate mofetil.^10^ Worsening liver injury as a complication of the liver biopsy was considered in our patient, but the findings on the liver biopsy specimen and the overall trajectory of the patient made this less likely.

We believe that nivolumab caused a cholestatic hepatic injury in our patient, and his presentation differs from most cases reported in the literature. Although he was treated with ceftriaxone for SBP during his hospitalization, he had presented with a mixed hepatocellular and cholestatic pattern of liver injury (before receiving ceftriaxone). It is very likely that the biopsy changes were because of the nivolumab exposure. The diverse clinical, biochemical, and histopathologic features of nivolumab-induced liver injury may suggest subtle differences in pathophysiology and warrant different strategies for the management of nivolumab-induced liver injury.

## DISCLOSURES

Author contributions: C. Anugwom wrote the manuscript. T. Leventhal revised the manuscript for intellectual content and is the article guarantor.

Financial disclosure: None to report.

Previous presentation: This case was presented at the American College of Gastroenterology Annual Scientific Meeting, October 25-30, 2019; San Antonio, Texas.

Informed consent was obtained from the patient's next of kin for this case report. All identifying information has been removed from this case report to protect patient privacy.
